# Challenges and Outcomes of Retinoblastoma Treatment in Ethiopia A Case of Jimma University Medical Center, Southwest Ethiopia

**DOI:** 10.4314/ejhs.v35i2.5

**Published:** 2025-03

**Authors:** Kumale Tolesa Daba, Diriba Fufa Hordofa, Aemero Abateneh Mengesha

**Affiliations:** 1 Department of Ophthalmology, Jimma University, Ethiopia; 2 Department of Pediatrics and Child Health, Jimma University, Ethiopia; 3 Department of Ophthalmology, Bahirdar University, Ethiopia

**Keywords:** Retinoblastoma, pediatric oncology, treatment outcomes, Ethiopia, healthcare disparities

## Abstract

**Background:**

Retinoblastoma, the most common intraocular malignancy in children, presents significant challenges globally, especially in low-resource settings like Ethiopia. This study aimed to assess the challenges and outcomes of retinoblastoma treatment at Jimma University Medical Center from October 2015 to September 2022.

**Methods:**

A retrospective study was conducted to evaluate the treatment outcomes of retinoblastoma patients. Statistical analysis was performed using SPSS Version 26, with variables summarized as frequencies, percentages, means, or medians.

**Results:**

A total of 43 children were treated from October 2015 to September 2022. The most common symptom recognized by caregivers was leucocoria (67.4%). Proptosis was the most frequent presenting symptom (53.5%), and delayed healthcare seeking was common. Diagnostic tools primarily included B-scan ultrasound (65.1%). Most children presented with advanced disease stages (Group D or E), requiring aggressive treatments such as intravenous chemotherapy (74.4%) and enucleation (23.8%). However, treatment adherence was poor, with high abandonment rates (55.8%) and incomplete chemotherapy cycles (81.3%). Metastasis occurred in 40% of patients, highlighting the aggressive nature of the disease. The mortality rate was 20.9%, mainly due to disease progression exacerbated by treatment interruptions. Challenges in follow-up and communication with remote patients further complicated outcomes assessment.

**Conclusion:**

The treatment outcomes for retinoblastoma were suboptimal, affected by delayed presentation and inadequate adherence to treatment. This emphasizes the critical need for improved early detection programs, enhanced treatment adherence strategies, and strengthened healthcare infrastructure to mitigate the impact of retinoblastoma and improve treatment outcome in resource-limited settings like Ethiopia.

## Introduction

Retinoblastoma, a malignant tumor arising from the retina, is the most common intraocular malignancy in children, affecting approximately 1 in 15,000 to 20,000 live births globally (1). This disease not only has profound medical consequences for the affected child but also imposes significant psychosocial and economic burdens on families and communities. These impacts vary across regions due to disparities in healthcare access and awareness.

In developed countries, early detection through systematic screening programs and advanced treatment modalities result in cure rates exceeding 95% (2). In contrast, in resource-limited settings, delayed diagnosis and limited access to healthcare facilities often lead to advanced disease at presentation, contributing to poorer outcomes (1). Recent advancements in multimodal treatments have improved survival rates in tertiary centers (3).

In Africa, where the incidence of retinoblastoma is higher, challenges in healthcare infrastructure and socio-economic factors exacerbate treatment delays (2). Studies from various African nations emphasize the need for improved diagnostic and treatment facilities, as well as supportive care services to enhance survival rates and reduce the psychosocial impact (4). Additionally, research on the abandonment of care across multiple African countries reveals systemic challenges (5).

Ethiopia exemplifies these challenges, with regional disparities in healthcare access, limited pediatric oncology services, and diagnostic resources leading to late-stage presentations of retinoblastoma (6). The untreated progression of retinoblastoma in such resource-poor settings underscores the urgent need for effective intervention strategies (7).

This study aimed to investigate the clinical characteristics, treatment modalities, and outcomes in 43 children diagnosed with retinoblastoma and treated at Jimma University Medical Center. By examining these factors within the Ethiopian context, the study aims to provide insights that could inform interventions and policy initiatives to improve retinoblastoma care and outcomes in Ethiopia and similar resource-constrained settings.

## Methods and Materials

**Study design**: This study was conducted at Jimma University Medical Center in Ethiopia from October 2015 to September 2022. A retrospective observational design was chosen to assess historical data related to treatment outcomes and challenges, which is appropriate for studying rare diseases like retinoblastoma in resource-limited settings.

**Participant selection**: The study included all children diagnosed with retinoblastoma and treated at Jimma University Medical Center during the study period. No exclusion criteria were applied based on age, gender, or disease stage, ensuring that all diagnosed cases were considered. This approach aimed to provide a comprehensive overview of the treatment outcomes and challenges faced by these patients.

**Data collection**: Medical records were reviewed to collect demographic information (age, gender, region of origin), clinical presentations (symptoms, diagnostic modality), nutritional status, disease staging (International Retinoblastoma Staging System), treatment modalities (chemotherapy, surgery), and treatment outcomes (response to therapy, survival status). Challenges encountered during treatment, such as delays in presentation, access to diagnostic tools, and resource limitations, were also documented.

**Statistical analysis**: Descriptive statistics were performed using SPSS Version 26 to summarize demographic characteristics, clinical presentations, treatment modalities, and outcomes. Categorical variables were presented as frequencies and percentages, while continuous variables were summarized using means with standard deviations or medians with interquartile ranges, as appropriate.

**Ethical considerations**: Ethical approval for the study was obtained from the Institutional Review Board (IRB) of Jimma University. Informed consent was waived due to the observational nature of the study, with all data anonymized to ensure patient confidentiality. The study adhered to the principles outlined in the Declaration of Helsinki.

**Limitations**: The limitations of the study include the small sample size and the retrospective nature of the research, which may affect the generalizability of the findings.

## Results

A total of 43 children with retinoblastoma were treated at Jimma University Medical Center between October 2015 and September 2022. The majority of patients (28, 65.1%) were from the Oromia region, followed by the Southwest Ethiopia region (9, 20.9%) and Gambella region (6, 14%). The age at presentation ranged from 3 to 132 months, with an average age of 67.5 months and a mean of 34.65 months (SD = 23.45).

Leucocoria was the most common symptom (67.4%), followed by red eye and proptosis (14% each). A delay in seeking healthcare was common, with 32.6% of patients presenting within 2-3 months of symptom onset, and 20.9% presenting after 12 months. Proptosis was the most frequent presenting symptom (53.5%). Visual acuity in the affected eye was poor at the time of presentation in nearly all cases (see Table 1)

**Table 1:** Visual Acuity status of retinoblastoma patients at JUMC between August 2016 and December 2022

Visual acuity	Right eye		Left eye	
		
	Frequency	Percent	Frequency	Percent
Fix and follow	19	45.2	12	28.6
No fix and follow	19	45.2	25	59.6
<20/400 (6/120)	4	9.6	1	2.3
20/50 (6/15) - 20/400 (6/120)			1	2.3
≥20/40 (6/12)			2	4.8
Unknown			1	2.3

B-scan ultrasound was the primary diagnostic tool used for 28 (65.1%) patients. Additional CT scans were required for 7 (16.3%) patients. Bilateral cases accounted for 7 (16.3%) of the total, with the left eye affected in 46.5% and the right eye in 37.2%. Nutritional status was assessed, with 62.8% of patients being well-nourished, while 37.2% exhibited varying degrees of malnutrition.

Treatment modalities included intravenous chemotherapy (74.4%) and enucleation (23.8%), with some patients receiving a combination of both. Palliative care was administered to one patient. Intravenous chemotherapy was the primary treatment for most patients, though treatment adherence was suboptimal. Only 18.7% of patients completed the recommended 8 cycles of chemotherapy(see table 2), and treatment abandonment occurred in 55.8% of cases.

**Table 2:** Number of total cycle of intravenous chemotherapy given to retinoblastoma patients at JUMC between August 2016 and December 2022

Number of total cycle of IV chemotherapy given	Frequency	Percent
1	7	21.9
2	5	15.6
3	4	12.5
2	3	9.4
6	4	12.5
7	1	3.1
8	6	18.7
10	2	6.3
Total	32	100.0

Metastasis was observed in 16 (40%) patients, predominantly affecting the skull and brain. Twenty-nine patients (66.7%) experienced treatment interruptions, resulting in 9 (20.9%) deaths, primarily due to disease progression.

## Discussion

This study highlights several critical challenges in the management of retinoblastoma in Ethiopia. The regional distribution of patients suggests geographic disparities in healthcare access, which could delay early diagnosis and treatment. Delays in seeking medical care, observed in over 20% of cases, echo findings from other African countries, where late presentation often contributes to worse outcomes (4).

The most common presenting symptom was leucocoria, which aligns with global patterns (1). However, the delay in diagnosis and advanced disease at presentation (51.2% with Group D and 62.7% with Group E disease) highlight the need for better screening and earlier intervention (10). Malnutrition was common among patients, which complicates treatment and recovery (9).

Treatment adherence was a major challenge, with significant numbers of patients abandoning care (55.8%) or not completing the prescribed chemotherapy cycles. These findings are consistent with reports from other African nations, where socioeconomic barriers and logistical difficulties contribute to treatment interruptions (13). The mortality rate was high (20.9%), and metastasis occurred in 40% of patients, underlining the aggressive nature of the disease in this cohort.

Efforts to improve retinoblastoma care in Ethiopia should focus on enhancing early detection, expanding healthcare infrastructure, and providing nutritional and psychosocial support for families. These interventions, combined with improved referral systems and follow-up, could significantly improve outcomes for children with retinoblastoma.

In conclusion, this study emphasizes the need for enhanced awareness, early diagnosis, and improved treatment adherence strategies to combat the challenges of retinoblastoma in Ethiopia. The high mortality rate is dictated by late presentation and treatment abandonment highlighting the need for health education on early detection and treatment compliance to improve the treatment outcome. Strengthening healthcare infrastructure and community-based support systems will be critical in addressing the barriers to care observed in this study. Additionally, further research and policy interventions targeting healthcare access and disease awareness are essential to reduce the burden of retinoblastoma in Ethiopia and similar resource-constrained settings.
